# Differences in Subjective and Objective Cognitive Decline Outcomes Are Associated with Modifiable Protective Factors: A 4-Year Longitudinal Study

**DOI:** 10.3390/jcm11247441

**Published:** 2022-12-15

**Authors:** Osamu Katayama, Sangyoon Lee, Seongryu Bae, Keitaro Makino, Ippei Chiba, Kenji Harada, Masanori Morikawa, Kouki Tomida, Hiroyuki Shimada

**Affiliations:** 1 Department of Preventive Gerontology, Center for Gerontology and Social Science, National Center for Geriatrics and Gerontology, 7-430 Morioka-cho, Obu City 474-8511, Aichi, Japan; 2 Japan Society for the Promotion of Science, Chiyoda 102-0083, Tokyo, Japan; 3 Department of Health Care and Science, Dong-A University, 37 Nakdong-daero 550, Saha-gu, Busan 49315, Republic of Korea; 4 Tohoku Medical Megabank Organization (ToMMo), Tohoku University, 2-1 Seiryo-machi, Aoba-ku, Sendai 980-8573, Miyagi, Japan

**Keywords:** subjective cognitive decline, objective cognitive decline, protective factors, community-dwelling older adults

## Abstract

Subjective cognitive decline (SCD) in older adults has been identified as a risk factor for dementia. However, the literature is inconsistent, and the underlying mechanisms are not well understood. We aimed to determine whether older adults with SCD had more modifiable protective factors against the risk of dementia and a lower risk of developing objective cognitive decline (OCD). We included 4363 older adults (71.7 ± 5.3 [mean ± standard deviation] years of age; 2239 women) from the National Center for Geriatrics and Gerontology Study of Geriatric Syndromes. SCD, OCD, and protective factors against dementia, such as lifestyle and activity, were assessed using interviews and objective cognitive-assessment tools. Based on initial cognitive status, participants were categorized into normal cognition, SCD-only, OCD-only, and both SCD and OCD groups. After 4 years, participants were classified as having either no impairment or mild or global cognitive impairment (i.e., OCD). Binomial logistic regression analyses were performed with the cognitive statuses of the groups at follow-up and baseline as the dependent and independent variables, respectively. After adjusting for potential confounding factors, we found that the SCD-only group had more modifiable protective factors against the risk of dementia than the OCD-only group. Community-dwelling older adults with normal cognition or those part of the SCD-only group had a lower risk of developing OCD during the 4-year follow-up, which may have been due to having more modifiable protective factors against the risk of dementia. Additionally, these factors may contribute to the inconsistencies in the literature on SCD outcomes.

## 1. Introduction

Subjective cognitive decline (SCD) may become increasingly important to clinicians in the future as more individuals seek medical care for cognitive decline, despite the lack of objective symptoms [1]. In 2014, researchers coined the term “SCD” [2] with two main characteristics: (1) a self-experienced persistent cognitive decline from a previously normal cognitive state, unrelated to an acute event; and (2) a normal performance in standardized cognitive-functioning tests used to classify mild cognitive impairment (MCI), the results of which were adjusted for age, sex, and education [1]. Population-based studies suggest that between 50% and 80% of older adults whose cognitive function tests are within normal limits report feeling a decline in cognitive function [3,4]. SCD characterizes the critical period between having normal cognitive function and experiencing cognitive decline. Additionally, according to the 2011 National Institute of Aging–Alzheimer’s Association criteria, SCD is classified as preclinical Alzheimer’s disease [5].

Longitudinal studies on SCD show that the condition is associated with a risk of developing objective cognitive decline (OCD), including MCI and dementia [6,7,8]. Characteristics that increase the risk of cognitive decline in SCD are referred to as “SCD plus” and include the following: subjective decline in memory, irrespective of function in other cognitive domains; onset of SCD within the past 5 years; onset of SCD at ages ≥ 60 years; concern of SCD; persistence of SCD over time; seeking medical help; and cognitive decline confirmed by an observer [1]. Most cases of SCD do not progress to dementia [1]. However, the factors that mitigate the transition from SCD to OCD are not clear, with inconsistent evidence in the literature. Growing awareness of brain health and Alzheimer’s disease in the general population is increasing the number of cognitively unimpaired individuals who are concerned about their reduced cognitive function, causing them to seek medical assistance [1]. Additionally, increased public awareness may be creating health awareness behaviors.

In a longitudinal study of changes in cognitive function in older adults with SCD, age, education, and occupation were protective factors against cognitive decline [9]. However, these factors are difficult to modify late in life. Therefore, it is possible that older adults with SCD have an increased awareness of dementia and may adopt healthy behaviors that include modifiable protective factors.

Therefore, we believe it is important to examine the factors that influence changes in cognitive function, including protective factors that are relatively modifiable even in later life. We hypothesize that community-dwelling older adults with SCD have more modifiable protective factors against the risk of dementia than older adults with OCD and that differences in SCD and OCD outcomes are associated with modifiable protective factors. We aimed to investigate this hypothesis through a 4-year longitudinal study.

## 2. Methods

### 2.1. Study Sample and Design

**Figure 1:**
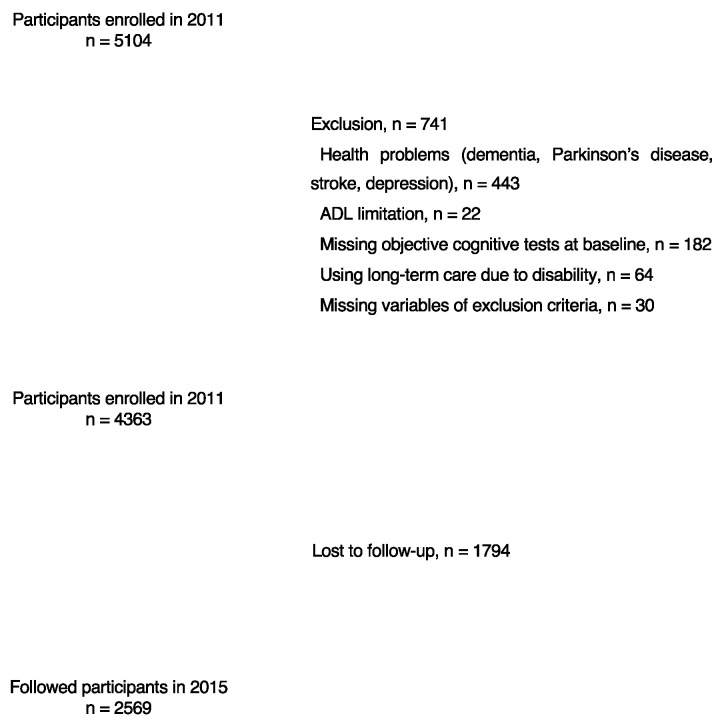
Flow diagram of sample selection. ADL, activities of daily living.

This was an observational, prospective, population-based cohort study involving adults enrolled in the National Center for Geriatrics and Gerontology Study of Geriatric Syndromes cohort study, which had the primary goal of establishing a screening system for validating evidence-based interventions to prevent geriatric syndromes [10]. A total of 5104 community-dwelling older adults participated in baseline assessments between August 2011 and February 2012, which included face-to-face interviews and measurements of physical and cognitive function. The inclusion criteria were as follows: (1) residents of Obu City; and (2) aged ≥ 65 years at the time of enrollment. The exclusion criteria were as follows: (1) health problems (dementia, Parkinson’s disease, stroke, or depression; *n* = 443), based on the information obtained by a qualified nurse during the face-to-face interviews to ensure that they had been diagnosed by a doctor; (2) inability to perform basic activities of daily living (ADLs), such as eating, grooming, bathing, and climbing up and down stairs (*n* = 22); (3) responses with missing objective cognitive test results at baseline (*n* = 182); (4) need for support or care due to a disability, as certified by the Japanese long-term care insurance system (*n* = 64); and (5) responses with missing exclusion criteria variables (*n* = 30). Based on these criteria, 741 participants were excluded and 4363 participants (mean age: 71.7 years, standard deviation [SD]: 5.3; 2239 women) were included in the analysis of baseline data. Our study did not include participants with developmental or intellectual disabilities or with acute psychosis. After excluding 1794 participants who were lost at follow-up, 2569 participants (mean age: 70.9 years, SD: 4.6; 1322 women) were included in the longitudinal analysis (Figure 1). All participants provided written informed consent prior to inclusion, and the study protocol was approved by the Ethics Committee of the National Center for Geriatrics and Gerontology (Approval Number: 1440-3).

### 2.2. Defining SCD and OCD

SCD was defined using the following criteria: (1) normal cognitive functioning on a neuropsychological assessment battery (i.e., scores > 1.5 SD units below age- and education-adjusted means); (2) the absence of OCD; and (3) a response of “Yes” to any one of the following four questions: (1) “Do you have any difficulty with your memory?”; (2) “Do you forget where you have left things more than you used to?”; (3) “Do you forget the names of close friends or relatives?”; and (4) “Do other people find you forgetful?” [11,12].

OCD was defined as MCI or global cognitive impairment (GCI). Cognitive screenings were conducted by trained staff using an iPad application called the National Center for Geriatrics and Gerontology–Functional Assessment Tool (NCGG–FAT) [13]. The tool comprises four domains: (1) memory (word list memory I [immediate recognition] and word list memory II [delayed recall]); (2) attention (a tablet version of Trail Making Test Part A); (3) executive function (a tablet version of Trail Making Test Part B); and (4) processing speed (a tablet version of the symbol digit substitution test). The tool has a high test–retest reliability and moderate to high criterion-related [13] and predictive validities [14] among community-dwelling older adults. As in a previous study, we reviewed available clinical, neuropsychological, and laboratory data with neurologists and neuropsychologists to identify participants with MCI [15]. MCI was diagnosed in individuals who exhibited cognitive impairment but were functionally independent in terms of ADLs [16]. In this study, MCI was defined as a decline in one or more domains. Global cognitive function was measured using the Mini-Mental State Examination (MMSE) [17], and an MMSE score of <24 points was determined as a cut-off for GCI [18]. The NCGG-FAT is excellent at assessing memory, attention, executive function, and processing speed, but it cannot assess global cognitive functions, such as orientation. Therefore, our institute evaluated objective cognitive functions using the NCGG-FAT and global cognitive functions using the MMSE. The results of both assessments were then used to determine MCI and GCI.

For all cognitive tests, established standardized thresholds were used in each corresponding domain to define impairment in the community-dwelling older adult population (scores of >1.5 SDs that specified age and educational means) [15,19]. Participants whose cognitive test scores were all >1.5 SD units above the mean were categorized as having normal cognition. Based on their baseline cognitive status, the participants were categorized into the following groups: normal cognition (neither SCD nor OCD); SCD-only; OCD-only; and both SCD and OCD.

### 2.3. Protective and Risk Factors for Dementia

The modifiable protective factors against and risk factors for dementia that were assessed included age, sex, years of education, self-reported chronic diseases, number of medications, nutritional status, body mass index (BMI), global cognitive function, physical functions, sleep duration, depressive symptoms, active lifestyle, and interpersonal interaction. Chronic diseases included heart disease, hypertension, and diabetes. Information on the number of medications was obtained through face-to-face interviews with nurses [20], and nutritional status was determined using albumin and total cholesterol levels from blood samples obtained more than four hours after the participants’ last meal. BMI was calculated using height and body weight, which was measured through a bioelectrical impedance analyzer (Tanita MC780A; Tanita Corp., Tokyo, Japan) [21]. Cognitive function was measured using the MMSE [17]. Physical functions were measured using grip strength and walking speed. Grip strength was defined as the maximum handgrip strength (kg) determined using a Smedley-type handheld dynamometer (GRIP-D; Takei Scientific Instruments Co., Ltd., Niigata, Japan). This parameter was tested under strictly standardized conditions, using the same device to avoid inter-observer and inter-device variability. In this assessment, participants were placed in the standing position with their elbows extended, and a measurement of the dominant hand’s grip strength was recorded [22]. Walking speed was measured in seconds using a stopwatch while participants walked on a flat and straight surface at a comfortable speed, and markers were used to indicate both the start and end of a 2.4 m walking path. Markers were also used to indicate the start and end of a 2 m section, and participants traversed this section at a comfortable pace before reaching the start marker of the timed path. To ensure a consistent walking pace on the timed path, participants were asked to continue walking for an additional 2 m past the end of the timed path [22]. In addition, sleep duration was calculated as the difference between the self-reported usual sleep and wake times of the participants [23]. Depressive symptoms were measured using the 15-item Geriatric Depression Scale (GDS) [24]. The GDS focuses on the functional and mood symptoms of depression rather than potentially misleading somatic features; thus, few somatic items were included in the scale. The 15-item GDS is a shortened, less time-consuming version of the 30-item GDS, specifically designed to screen depression in older adults.

To complete the questionnaire of ADLs and instrumental ADLs, the participants were required to respond with “yes” or “no” to each of the ten questions about different components of an active lifestyle, namely physical, cognitive, and social activities, as well as interpersonal interaction. In this questionnaire, the question used to measure physical activity was: (1) “Do you have any hobbies or sports activities”? The questions used to measure cognitive activity were the following: (2) “Do you read books or newspapers”; (3) “Do you drive a car”; (4) “Do you use a personal computer”; (5) “Do you engage in activities that use your brain (shogi, learning, etc.)”; and (6) “Do you operate a video/DVD player”? The questions used to measure social activity were the following: (7) “Are you a board member or secretary of a neighborhood association, senior citizens’ club, or non-profit organization”; and (8) “Do you engage in any activities related to environmental beautification (e.g., cleaning up parks)”? The questions used to measure interpersonal interaction were the following: (9) “Do you sometimes visit your friends”; and (10) “Do you have a friend to call”?

To complete our study questionnaire, participants were required to respond with “yes” or “no” to 10 questions regarding different components of an active lifestyle, namely physical, cognitive, and social activities as well as interpersonal interactions. We categorized the protective factors into binary values and counted the number of protective factors possessed by each group, as follows. The cut-off point for BMI was 25.0 kg/m^2^, indicative of obesity [25]; for years of education, it was was 10 years [26]; for the GDS score, it was 6, considered to indicate depressive symptoms [27]; for grip strength, it was 28 kg for men and 18 kg for women, according to the Asian Working Group for Sarcopenia, 2019 [28]; for walking speed, it was 1.0 m/s [28]; and for sleep duration, it was from 4 to 10 h, or less than 4 or more than 10 h [29].

### 2.4. Statistical Analysis

Pearson’s chi-squared tests and one-way analysis of variance (ANOVA) were used to examine the differences among the baseline characteristics of the normal cognition, SCD-only, OCD-only, and both SCD and OCD groups. Adjusted standardized residuals > 1.96 indicated *p* < 0.05. We categorized protective factors into binary values and compared the number of protective factors possessed among the four groups using ANOVA. Furthermore, based on 4-year follow-up assessments, participants were classified into two groups: those with MCI or GCI (i.e., OCD) and those without. A binomial logistic regression analysis was performed with the presence or absence of OCD at the follow-up assessment as the dependent variable and the baseline cognitive status as the independent variable. After using unadjusted models, we adjusted the covariates of the protective factors against and the risk factors for dementia (i.e., age, sex, years of education, self-reported chronic diseases, number of medications, nutritional status, BMI, cognitive function, physical functions, sleep duration, GDS, active lifestyle, and interpersonal interaction), with the data presented as odds ratios (ORs) with 95% confidence intervals (CIs). We divided the SCD-only group into two groups, one with more protective factors and the other with fewer protective factors, based on the median number of protective factors possessed, in order to explore whether having more SCD affects future outcomes in the SCD-only group as a sub-analysis. In addition, we used binomial logistic regression analysis to examine whether being in the group with fewer protective factors is associated with future OCD. The significance level was set at *p* < 0.05, and all analyses were performed using the International Business Machines (IBM) Statistical Package for the Social Sciences version 28.0 (IBM Corp., Armonk, NY, USA).

## 3. Results

Table 1 shows the baseline characteristics of the participants who participated in the follow-up survey and those who were lost at follow-up. Baseline characteristics of the participants who participated in the follow-up survey and those who were lost at follow-up showed significant differences in almost all variables. (*p* < 0.05).

At baseline, the normal cognition, SCD-only, OCD-only, and both SCD and OCD groups comprised 955 (21.9%), 2044 (46.8%), 386 (8.8%), and 978 (22.4%) participants, respectively. Table 2 shows the baseline characteristics of the cognitive status groups. Based on cognitive status, significant differences were observed in the characteristics of the four groups with respect to sex, except for chronic diseases and BMI (*p* < 0.05). The normal cognition and SCD-only groups were characterized by long years of education, maintained physical functioning, active lifestyles (i.e., physical, cognitive, and social activities), interpersonal interactions, and sleep durations (Table 2). The baseline characteristics of the study participants with and without OCD at follow-up, as well as those who dropped out of the study, were also compared. The dropout group was older, took more medications, had lower albumin levels, had decreased physical functioning, and possessed fewer protective factors against dementia (*p* < 0.05). We categorized protective factors into binary values and compared the number of protective factors possessed among the four groups. We found that the normal cognition group and the SCD-only group possessed significantly more protective factors than the other groups (*p* < 0.01). Furthermore, in our exploratory sub-analysis, we divided the SCD-only group into two groups, one with more protective factors and the other with fewer protective factors, based on the median value. Binomial logistic regression analyses showed that being in the group with fewer protective factors was significantly associated with the future development of OCD. The risk for developing OCD at follow-up was 1.65 (95% CI, 1.21–2.25; *p* = 0.002). The results also showed that, among the protective factors, personal computer use was most strongly associated with the development of OCD. After adjustment, the risk for developing OCD at follow-up was 1.66 (95% CI, 1.14–2.42; *p* = 0.009).

Table 3 shows the ORs and 95% CIs from the unadjusted and adjusted binomial logistic regression analyses. The following variables were adjusted for: age; sex; years of education; self-reported chronic diseases; number of medications; BMI; albumin; total cholesterol; MMSE score; grip strength; walking speed; sleep duration; GDS score; active lifestyle; and interpersonal interaction. After adjustment, the risks for developing OCD at follow-up were 1.08 (95% CI, 0.80–1.45; *p* = 0.608), 4.00 (95% CI, 2.64–6.07; *p* < 0.001), and 3.12 (95% CI, 2.22–4.37; *p* < 0.001) for the SCD-only, OCD-only, and SCD and OCD groups, respectively.

## 4. Discussion

As hypothesized, community-dwelling older adults with SCD had more modifiable protective factors against the risk of dementia and a lower risk of progression to OCD compared to older adults with OCD. Furthermore, the prevalence of SCD among the participants in our study was similar to that of previous population-based studies, with the slightly lower value in this study potentially being due to the younger mean age of our study participants [3]. 

Interestingly, we found that the SCD-only group was not associated with progression to OCD. The normal cognition and SCD-only groups were characterized by having more modifiable protective factors against dementia than the OCD-only and both SCD and OCD groups, including more years of education, maintained physical functioning, active lifestyles, and interpersonal interactions. Participants in the normal cognition and SCD-only groups had more years of education than those in the OCD group. Older adults with SCD have been reported to have more years of education than older adults with MCI [30]. On the other hand, some reports state no statistical difference in the years of education between participants with SCD and those with MCI [31]. Further studies may be needed to clarify this association. Nevertheless, higher educational attainment in childhood and throughout life is associated with a lower risk of dementia [20,32], and people with higher cognitive functions may seek out cognitively stimulating activities and education [33]. In our study, participants with normal cognition and those in the SCD-only group maintained active lifestyles, particularly in terms of physical functioning, physical activity, and cognitive activity. These two groups also engaged in interpersonal interactions, such as social participation, suggesting that more years of education may be related to an active lifestyle. A recent study showed that education was a predictor of cognitive function in older adults [9]. In addition, differences in social networks, including interactions with others, were found between older adults with normal cognitive function and those with OCD [34]. Significant differences between groups were also found regarding MMSE scores and sleep duration. Both insufficient and excessive sleep duration have been significantly associated with cognitive decline [29,35]. The SCD and OCD group slept longer and had lower MMSE scores compared to those in the other groups, consistent with previous results [29,35]. However, in this study, interviewing participants regarding the number of times they were awake during the night was not possible. Therefore, sleep duration may have been overestimated if insomnia symptoms were present.

Behavioral changes necessary for participants to engage in healthy behaviors [36] typically occur in stages. Early in the process of adopting a healthy behavior, increasing an individual’s interest from a state of indifference is important. Globally, the number of patients with dementia and general interest in SCD is increasing [1]. As such, older adults with SCD may take action to improve their health. For most individuals with SCD, a study suggests assuring them that their condition will not transition to OCD in the near future and providing strategies to support brain health are vital [1]. In particular, these strategies should include modifiable risk factors for dementia, control of hypertension and diabetes, treatment of mood disorders, physical exercise, weight control, a Mediterranean-style diet, smoking cessation, cognitive and social engagement activities, high-quality sleep, stress reduction, and the use of hearing aids [20]. The results of this study supported those of previous studies which suggested an active lifestyle as a protective factor against dementia in older adults with SCD [1,20]. Further, among the SCD-only group, those with a higher number of modifiable protective factors had a lower risk of developing future OCD. Specifically, our results suggest that the inconsistency in the literature on SCD outcomes may be, in part, due to the fact that certain older adults with SCD have more modifiable protective factors against the risk of dementia than others [1,6,7,8,9]. SCD, a preclinical stage of Alzheimer’s disease, is the critical period between normal cognitive function and the development of cognitive decline [5]. Nevertheless, the identified association between SCD and modifiable protective factors against dementia may provide important information to prevent MCI and dementia.

The strength of this study was its longitudinal design in analyzing the factors associated with OCD development. In particular, it employed a large cohort of community-dwelling older adults who were categorized based on their cognitive status and into normal cognition, SCD-only, OCD-only, and both SCD and OCD groups, focusing on the modifiable protective factors against and risk factors for dementia. However, this study had some limitations. First, participants were not randomly recruited, and approximately 41% of them dropped out at follow-up, which may have led to an underestimation of OCD at follow-up. Second, the cognitive function test for diagnosing MCI used to define OCD consisted of one test per domain. Future studies may increase the validity of the test results by adding additional tests per domain. Third, as in previous studies [6,30], this study had a 4-year longitudinal design; however, similar studies have used 6.8- to 8-year longitudinal designs [37,38,39]. In addition, different sample characteristics, such as age, in each study may have influenced the differences in the respective results. In this regard, longitudinal testing on young or middle-aged adults in future studies may be required. Fourth, we were unable to evaluate the frequency and intensity of each activity and their interaction in detail. In the future, we will focus on the optimal frequency and intensity of exercise to prevent OCD in older adults with SCD. Finally, we were unable to examine the genetic and socioeconomic confounding factors related to the risk factors of dementia, which may be examined in future studies.

Compared with the other groups, community-dwelling older adults with SCD had more modifiable protective factors against the risk of dementia, including more years of education, physical functioning, active lifestyle, and interpersonal interactions. Furthermore, they had a lower future risk of progression to OCD. Our longitudinal data suggested that the presence of modifiable protective factors against the risk of dementia may contribute to the inconsistency in the literature on SCD outcomes.

## Figures and Tables

**Table 1:** Baseline characteristics of study participants by follow-up status.

	Total	Missing	Followed Participants	Lost at Follow-Up		
	*n* = 4363	Number	*n* = 2569	*n* = 1794	*p*	Cohen’s d
Age, y	71.7 ± 5.3	0	70.9 ± 4.6	72.9 ± 6.1	<0.001 *	−0370
Sex, female %	2239 (51.3)	0	1322 (51.5)	917 (51.1)	0.823	
Heart disease, yes %	699 (16.0)	0	401 (15.6)	298 (16.6)	0.375	
Hypertension, yes %	1963 (45.0)	0	1086 (42.3) ^§^	876 (48.8) ^‡^	<0.001 ^†^	
Diabetes, yes %	573 (13.1)	0	313 (12.2) ^§^	260 (14.5) ^‡^	0.026 ^†^	
Medications, number	1.9 ± 2.0	0	1.8 ± 1.9	2.1 ± 2.1	<0.001 *	−0.164
BMI, kg/m^2^	23.4 ± 3.1	25	23.4 ± 2.9	23.4 ± 3.3	0.462	−0.023
Albumin, mg/dL	4.3 ± 0.3	30	4.3 ± 0.2	4.3 ± 0.3	<0.001 *	0.193
Total cholesterol, mg/dL	208.8 ± 33.5	30	209.4 ± 32.4	207.8 ± 35.0	0.120	−0.012
Education, y	11.4 ± 2.5	0	11.7 ± 2.5	11.0 ± 2.5	<0.001 *	0.199
MMSE score	26.5 ± 2.4	0	26.8 ± 2.3	26.1 ± 2.5	<0.001 *	0.216
Word list memory, composite score	11.2 ± 2.9	0	11.6 ± 2.7	10.6 ± 3.0	<0.001 *	0.365
TMT-A, seconds	20.9 ± 6.2	0	19.9 ± 5.1	22.2 ± 7.3	<0.001 *	−0.374
TMT-B, seconds	42.5 ± 17.1	0	39.6 ± 14.9	46.5 ± 19.1	<0.001 *	−0.410
SDST, score	38.6 ± 8.1	0	40.1 ± 7.4	36.4 ± 8.5	<0.001 *	0.480
Grip strength, kg	27.0 ± 7.9	192	27.6 ± 7.8	26.2 ± 8.0	<0.001 *	0.178
Walking speed, m/sec	1.2 ± 0.2	6	1.2 ± 0.2	1.2 ± 0.2	<0.001 *	0.421
Sleep duration, minutes	461.9 ± 73.8	0	455.4 ± 67.9	471.2 ± 80.6	<0.001 *	−0.216
GDS, score	2.7 ± 2.5	13	2.4 ± 2.3	3.2 ± 2.6	<0.001 *	−0.299
Do you have any hobbies or sports activities? no (%)	1138 (26.1)	8	523 (20.4) ^§^	615 (34.4) ^‡^	<0.001 ^†^	
Do you read books or newspapers? no (%)	168 (3.9)	1	71 (2.8) ^§^	97 (5.4)^‡^	<0.001 ^†^	
Do you drive a car? no (%)	1218 (27.9)	5	593 (23.1) ^§^	625 (34.9) ^‡^	<0.001 ^†^	
Do you use a personal computer? no (%)	2852 (65.4)	1	1544 (60.1) ^§^	1308 (72.9) ^‡^	<0.001 ^†^	
Do you engage in activities that use your brain (shogi, learning, etc.)? no (%)	2178 (50.0)	9	1205 (47.0) ^§^	973 (54.4) ^‡^	<0.001 ^†^	
Do you operate a video/DVD player? no (%)	2022 (46.4)	3	1120 (43.6) ^§^	902 (50.3) ^‡^	<0.001 ^†^	
Are you a board member or secretary of a neighborhood association, senior citizens’ club, or non-profit organization? no (%)	2940 (67.5)	8	1613 (62.9) ^§^	1327 (74.1) ^‡^	<0.001 ^†^	
Do you engage in any activities related to environmental beautification (e.g., cleaning up parks)? no (%)	2962 (67.9)	1	1643 (64.0) ^§^	1319 (73.6) ^‡^	<0.001 ^†^	
Do you sometimes visit your friends? no (%)	551 (12.6)	4	272 (10.6) ^§^	279 (15.6) ^‡^	<0.001 ^†^	
Do you have a friend to call? no (%)	253 (5.8)	10	144 (5.6)	109 (6.1)	0.504	
Protective factors, *n*	13.4 ± 2.8	194	14.0 ± 2.6	12.6 ± 2.9	<0.001 *	0.493

* *p*-values reported from Student’s *t*-test. ^†^ *p*-values obtained by Pearson’s chi-squared test. ^‡^ Statistically significant association was determined by adjusted standardized residual > 1.96 (*p* < 0.05). ^§^ Statistically significant association was determined by adjusted standardized residual < −1.96 (*p* < 0.05).

**Table 2:** Comparisons of potential confounders and cognitive status at baseline.

	Normal Cognition	SCD-Only	OCD-Only	SCD and OCD			
	*n* = 955	*n* = 2044	*n* = 386	*n* = 978	*p*	η^2^	Post-Hoc
Age, y	71.1 ± 4.8	71.4 ± 5.2	72 ± 5.4	73 ± 5.8	<0.001 *	0.018	N < O < SO
Sex, female %	509 (53.3)	1083 (53.0) ^‡^	176 (45.6) ^§^	471 (48.2) ^§^	0.005		
Heart disease, yes %	122 (12.8) ^§^	351 (17.2)	46 (11.9) ^§^	180 (18.4) ^‡^	0.585		
Hypertension, yes %	423 (44.3)	893 (43.7)	176 (45.6)	470 (48.1) ^‡^	0.866		
Diabetes, yes %	108 (11.3)	281 (13.7)	41 (10.6)	143 (14.6)	0.058		
Medications, number	1.7 ± 1.8	1.9 ± 2.0	1.9 ± 1.9	2.2 ± 2.1	<0.001 *	0.007	N, S, O < SO
BMI, kg/m^2^	23.4 ± 3.1	23.3 ± 3.1	23.8 ± 3.3	23.4 ± 3.1	0.084	0.002	
Albumin, mg/dL	4.4 ± 0.3	4.3 ± 0.3	4.3 ± 0.2	4.3 ± 0.3	<0.001 *	0.008	SO < S < N
Total cholesterol, mg/dL	211.6 ± 33.4	209.4 ± 33.5	206.3 ± 30.9	205.5 ± 34.4	0.009 *	0.004	SO < S; O, SO < N
Education, y	11.6 ± 2.4	11.7 ± 2.5	11.1 ± 2.4	10.9 ± 2.5	<0.001 *	0.018	O, SO < N, S
MMSE score	27.2 ± 1.9	27.3 ± 1.8	24.6 ± 2.5	24.8 ± 2.6	<0.001 *	0.241	O, SO < N, S
Word list memory, composite score	12.0 ± 2.4	11.9 ± 2.5	9.8 ± 2.9	9.5 ± 3.0	<0.001 *	0.150	O, SO < N, S
TMT-A, seconds	19.1 ± 3.9	19.3 ± 4.3	24.2 ± 7.6	24.7 ± 8.3	<0.001 *	0.160	N, S < O, SO
TMT-B, seconds	35.7 ± 9.6	36.1 ± 10.2	55.0 ± 19.9	57.3 ± 20.7	<0.001 *	0.315	N, S < O, SO
SDST, score	41.1 ± 6.9	40.7 ± 7.1	34.5 ± 7.9	33.2 ± 8.1	<0.001 *	0.177	SO < O < N, S
Grip strength, kg	27.1 ± 7.7	27.3 ± 7.9	27.4 ± 8.3	26.4 ± 8.1	0.041 *	0.002	SO < S
Walking speed, m/sec	1.2 ± 0.2	1.2 ± 0.2	1.2 ± 0.2	1.1 ± 0.2	<0.001 *	0.029	SO < O < N, S
Sleep duration, minutes	459.8 ± 67.8	457.3 ± 69.2	464.9 ± 84.9	472.4 ± 82.5	<0.001 *	0.007	N, S < SO
GDS, score	1.8 ± 1.9	2.9 ± 2.5	2.2 ± 2.1	3.5 ± 2.7	<0.001 *	0.060	N < O < S < SO
Do you have any hobbies or sports activities? no (%)	222 (23.3) ^§^	482 (23.6) ^§^	131 (33.9) ^‡^	303 (31.0) ^‡^	<0.001 ^†^		
Do you read books or newspapers? no (%)	26 (2.7) ^§^	60 (2.9) ^§^	16 (4.1)	66 (6.7) ^‡^	<0.001 ^†^		
Do you drive a car? no (%)	252 (26.4)	517 (25.3) ^§^	119 (30.9)	330 (33.7) ^‡^	<0.001 ^†^		
Do you use a personal computer? no (%)	606 (63.5)	1233 (60.4) ^§^	286 (74.1) ^‡^	727 (74.3) ^‡^	<0.001 ^†^		
Do you engage in activities that use your brain (shogi, learning, etc.)? no (%)	413 (43.3) ^§^	980 (48.0) ^§^	190 (49.2)	595 (61.0) ^‡^	<0.001 ^†^		
Do you operate a video/DVD player? no (%)	413 (43.2) ^§^	879 (43.0) ^§^	203 (52.7) ^‡^	527 (53.9) ^‡^	<0.001 ^†^		
Are you a board member or secretary of a neighborhood association, senior citizens’ club, or non-profit organization? no (%)	631 (66.1)	1318 (64.6) ^§^	272 (70.6)	719 (73.7) ^‡^	<0.001 ^†^		
Do you engage in any activities related to environmental beautification (e.g., cleaning up parks)? no (%)	665 (69.6)	1327 (65.0) ^§^	282 (73.1) ^‡^	688 (70.3)	0.001 ^†^		
Do you sometimes visit your friends? no (%)	107 (11.2)	236 (11.6) ^§^	58 (15.0)	150 (15.4) ^‡^	0.006 ^†^		
Do you have a friend to call? no (%)	41 (4.3) ^§^	101 (4.9) ^§^	32 (8.4) ^‡^	79 (8.1) ^‡^	<0.001 ^†^		
Protective factors, *n*	13.8 ± 2.6	13.7 ± 2.7	13.0 ± 2.8	12.5 ± 2.8	<0.001 *	0.038	SO < O < N, S

* *p*-values reported from one-way ANOVA. ^†^ *p*-values obtained by Pearson’s chi-squared test. ^‡^ Statistically significant association was determined by adjusted standardized residual > 1.96 (*p* < 0.05). ^§^ Statistically significant association was determined by adjusted standardized residual < −1.96 (*p* < 0.05). SCD, subjective cognitive decline; OCD, objective cognitive decline; BMI, body mass index; MMSE, Mini-Mental State Examination; TMT, Trail Making Test; SDST, symbol digit substitution test; GDS, 15-item Geriatric Depression Scale; y, years; N, normal cognition; S, SCD-only; O, OCD-only; SO, SCD and OCD.

**Table 3:** Binomial logistic regression analysis with presence of OCD at follow-up as a dependent variable.

	Crude Model			Adjusted Model		
	OR	95% CI	*p*	OR	95% CI	*p*
Normal cognition	1.00			1.00		
SCD-only	1.09	0.84–1.43	0.520	1.08	0.80–1.45	0.608
OCD-only	4.80	3.33–6.91	<0.001	4.00	2.64–6.07	<0.001
SCD and OCD	4.45	3.34–5.93	<0.001	3.12	2.22–4.37	<0.001

Adjusted model is adjusted for age, sex, years of education, self-reported chronic diseases, number of medications, nutritional status, body mass index, cognitive function, physical functions, sleep duration, depressive symptoms, active lifestyle, and interpersonal interaction. CI, confidence interval; OCD, objective cognitive decline; OR, odds ratio; SCD, subjective cognitive decline.

## Data Availability

The datasets used and/or analyzed during the present study are available from the corresponding author on reasonable request.
